# Mediastinal tuberculosis following descending necrotizing mediastinitis: A case report

**DOI:** 10.1002/rcr2.957

**Published:** 2022-05-01

**Authors:** Akira Matsumoto, Toshihiko Soma, Tsuyoshi Shoji, Hiromichi Katakura

**Affiliations:** ^1^ Department of Thoracic Surgery Otsu Red Cross Hospital Nagara, Otsu Shiga Japan

**Keywords:** descending necrotizing mediastinitis, extrapulmonary tuberculosis, thoracic surgery

## Abstract

Tuberculosis is a disease that causes latent infection and is sometimes activated by a variety of factors. Descending necrotizing mediastinitis (DNM) is a serious disease caused by spreading oropharyngeal infection. We present a case of mediastinal tuberculosis following mediastinal dissection and antibiotic therapy for DNM. A 62‐year‐old man was admitted to the hospital with an increasing mass in the right mediastinum during outpatient follow‐up after surgical drainage and antibiotic treatment for idiopathic cervical abscess and left DNM caused by oral bacteria. The patient underwent right mediastinal abscess dissection 4 months after the last surgery. As a result of culture tests, no general bacteria but *Mycobacterium tuberculosis* was detected. Anti‐tuberculosis treatment was continued for 9 months, and the patient has progressed without any recurrence of infection. The possibility of relapse of tuberculosis should always be considered in patients with unexplained masses.

## Graphical abstract

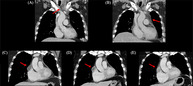

## INTRODUCTION

*Mycobacterium tuberculosis* is known to cause asymptomatic latent tuberculosis infection. It can sometimes progress to active tuberculosis, and the likelihood of the progression is determined by bacterial, host and environmental factors.^1^ It most often affects the lungs, but the disease may also involve other parts of the body.^2^

Descending necrotizing mediastinitis (DNM) is an acute mediastinitis caused by spreading oropharyngeal infection. It is a rare but life‐threatening condition and requires urgent drainage when diagnosed. Multiple surgical drainage is sometimes required.^3^

We present a case of mediastinal tuberculosis diagnosed by an enlarging abscess in the right mediastinum, after left mediastinal dissection and antibiotic therapy for DNM.

## CASE REPORT

**FIGURE 1:**
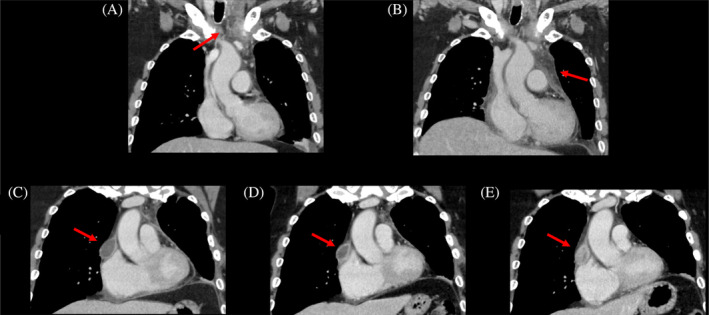
Coronal thorax computed tomography (CT) scans show the abscess from the neck to the mediastinum at hospital admission (A) and show further progression of the infection with mediastinal enlargement on the third day in the hospital (B). Improvement of the left mediastinal abscess was observed after debridement and antibiotics treatment, but the contralateral mediastinal abscess remained on CT at 2 weeks after surgery (C). CT showed slight shrinkage of the mass 4 weeks after surgery (D), but it increased 13 weeks after surgery (E).

A 62‐year‐old man was admitted with neck pain, hoarseness and high inflammatory response. An enhanced computed tomography (CT) scan (Figure 1A) showed fluid from the neck to the mediastinum, and the patient was diagnosed with idiopathic cervical abscess and left DNM. He had a past history of hypertension, hyperlipidaemia and abdominal aortic aneurysm; had no immunosuppressive disorders; and had received the BCG vaccine when he was a child.

Broad‐spectrum antibiotic therapy with meropenem was initiated. However, on the third day in the hospital, CT showed mediastinal enlargement (Figure 1B), and the patient underwent cervical incision and lavage and left mediastinal abscess dissection. During the surgery, the patient suffered a total of 1564 ml of bleeding due to pulmonary artery injury, which required a blood transfusion and 4 days of postoperative care in the intensive care unit. One week after surgery, *Parvimonas micra* was identified from culture tests. Depending on the drug sensitivity, the antibiotic was changed to sulbactam/ampicillin and continued for 3 weeks. CT at 2 weeks after surgery (Figure 1C) showed improvement of the left mediastinal abscess and a mass in the contralateral mediastinum, which had slightly shrunk on CT 4 weeks after surgery (Figure 1D). After changing the antibiotic to oral amoxicillin/clavulanate, the patient was discharged.

The patient was continued to be treated with oral antibiotics on an outpatient basis, but CT showed an enlarging mass in the contralateral mediastinum 13 weeks after surgery (Figure 1E). Although the patient was readmitted to the hospital and treated with intravenous antibiotics with sulbactam/ampicillin for 3 weeks, the mass did not improve. The patient underwent right mediastinal abscess dissection by video‐assisted thoracoscopic surgery. As a result of culture tests, no general bacteria but *M*. *tuberculosis* was detected, and the diagnosis of mediastinal tuberculosis was made. Four‐drug combination therapy with anti‐tuberculosis drugs was continued for 9 months, and the patient has progressed without any recurrence of mediastinal mass and any symptoms.

## DISCUSSION

Tuberculosis is a disease that causes latent infection and is sometimes activated. It is impossible to directly diagnose latent tuberculosis infection, so it is diagnosed by response to in vivo or in vitro stimulation by *M*. *tuberculosis* antigens using the tuberculin skin test or interferon‐γ release assays (IGRAs). The widely recognized risk factors for progression of latent infection include suppression of cellular immunity such as HIV infection, renal failure and environmental exposure such as silicosis.^1^

Tuberculosis sometimes affects sites other than the lungs. The common extrapulmonary tuberculosis are tuberculosis pleurisy, bronchial tuberculosis, tuberculosis lymphadenitis of the neck and tuberculosis meningitis, but the lesions are diverse. In addition, diagnosis of extrapulmonary tuberculosis is sometimes difficult due to the non‐specificity of clinical symptoms and imaging findings and the difficulty of culture tests.^2^ Mediastinal granuloma and fibrosing mediastinitis are common manifestations of mediastinal tuberculosis.^4^ These are chronic mediastinitis that occur late in chronic granulomatous lymphadenitis in the mediastinum with tuberculosis.^5^ It is rare for tuberculosis to cause acute mediastinal abscess.

DNM is a rare but life‐threatening disease caused by spreading oropharyngeal infection. It requires early diagnosis and aggressive surgical drainage. Multiple surgical drainage is sometimes required.^3^

In our case, the enlarging mediastinal mass on the opposite side of the surgery was observed after changing the treatment for DNM to oral antibiotics. Recurrence of contralateral DNM was most likely suspected, and the treatment plan of surgical drainage would not have changed. However, antimicrobial culture and pathology should have been performed at the time of initial surgery, and the tuberculin skin test or IGRAs should have been performed before reoperation. Although DNM is a serious disease and requires urgent attention, it reaffirmed the importance of differential diagnosis in infectious diseases. Unfortunately, we can only make a hypothesis because we have not performed pathology and initial antimicrobial culture tests. We presumed that *M*. *tuberculosis* latently infecting soft tissues or lymph nodes in the mediastinum and that critical postoperative conditions triggered the reactivation of tuberculosis.

In conclusion, tuberculosis can be activated following DNM in some patients. We reaffirmed the importance of considering the possibility of tuberculosis even in extrapulmonary masses.

## CONFLICT OF INTEREST

None declared.

## AUTHOR CONTRIBUTION

Akira Matsumoto and Tsuyoshi Shoji wrote the manuscript, which was then reviewed by all co‐authors.

## ETHICS STATEMENT

The authors declare that appropriate written informed consent was obtained for the publication of this manuscript and accompanying images.

## Data Availability

The data that support the findings of this study are available from the corresponding author upon reasonable request.
